# The evaluation of decision-to-delivery interval in category-1 emergency cesarean section: a report of six cases

**DOI:** 10.1186/s40981-022-00523-6

**Published:** 2022-04-26

**Authors:** Atsushi Kotera

**Affiliations:** Department of Anesthesiology, Kumamoto City Hospital, 4-1-60, Higashimachi, Higashi-ku, Kumamoto City, Kumamoto, 862-8505 Japan

**Keywords:** Category-1 emergency cesarean section, Decision-to-delivery interval, General anesthesia

## Abstract

**Background:**

In category-1 emergency cesarean section, decision-to-delivery interval (DDI) is an important indicator for evaluating the quality of maternity care. I thus evaluated DDI and neonatal outcome in category-1 emergency cesarean section in our institution.

**Case presentation:**

I collected data from the six patients undergoing category-1 emergency cesarean section performed between October 1, 2019, and December 31, 2021. The average age and gestational age were 32 years old (range, 21–42) and 34 weeks (range, 26–40), respectively. Three patients suffered from abruptio placenta and the others fetal distress. All the surgeries were performed under general anesthesia, and the average DDI was 21 min (range, 10–29). The morality was 25% in neonates whose gestational ages of >35 weeks, and that was 67% in neonates whose gestational ages of <27 weeks.

**Conclusions:**

DDI was achieved within 30 min in all the patients, and the mortality of neonate might depend on gestational age.

## Background

When the cesarean section (C/S) is done urgently for an immediate life-threatening condition of the fetus or mother, it is defined as category-1 emergency C/S [1], and guidelines recommend that decision-to-delivery interval (DDI) should be within 30 min [2]. DDI is an important indicator for evaluating the quality of maternity care [3]; however, in real clinical settings, to achieve DDI within 30 min is challenging. This study was aimed to evaluate DDI and neonatal outcome in catergory-1 emergency C/S in our institution.

## Patients and methods

### Patients

The approval for this study (approval no.605) was provided by Ethical Committee of Kumamoto City Hospital, Kumamoto, Japan, on January 19, 2022. Patients who underwent category-1 emergency C/S performed between October 1, 2019, and December 31, 2021, were eligible for this study. I published the details of this study on the homepage of Kumamoto City Hospital (Opt-out method).

### Data collection

Data were collected from the medical charts. Concerning a pregnant woman, I extracted age, gestation age, gravidity, height, weight, indication for C/S, single or twin pregnancy, underlying co-morbidities, inpatient or outpatient, and outcome. I also extracted the times of request for transfer to our institution, arrival at our institution, decision for C/S, arrival at the operating theater, start of the surgery, delivery of the neonate, end of the surgery, DDI, intraoperative blood loss, fluid infusion, and urine output. Concerning a neonate, I extracted fetal heart rate (FHR) on the arrival at our institution, Apgar score, weight, arterial cord blood tests, and outcome.

### Obstetric emergency call system in our institution

**Fig. 1:**
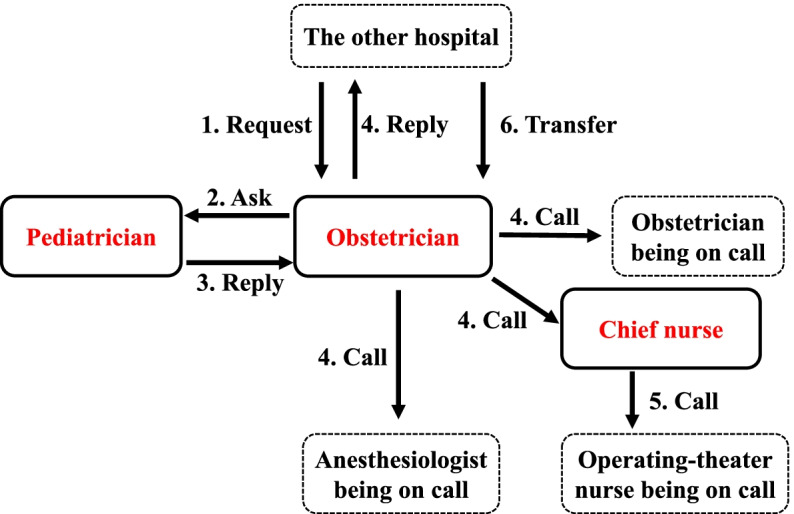
The flow chart of our obstetric emergency call system is demonstrated. Staff members who are staying in our hospital on a 24-h basis are circled by solid frame, and those who are on call duty are circled by a dotted frame

Our obstetric emergency call system is shown (Fig. 1). One obstetrician and one pediatrician are on duty in our hospital on a 24-h basis. However, another obstetrician, one anesthesiologist, and two operating-theater nurses are on-call duty, and they are not necessarily present in our hospital on a 24-h basis. When a transfer of a patient is requested from the other hospital to the obstetrician being on duty, the obstetrician calls anesthesiologist, another obstetrician, and operating-theater nurses who are on call as early as possible. Our goal is that the staffs being on-call can arrive at our hospital before a patient is coming to our hospital, and an over-triage is permitted in our call system.

## Results

During the study period, six cases of category-1 emergency C/S were undergone. Their clinical characteristics were shown in Table 1. The average age was 32 years old (range, 21–42), and the average gestational age was 34 weeks (range, 26–40). Three patients had abruptio placenta and the others fetal distress. One patient is pregnant with twins. The average FHR on the arrival at our institution was 71 beats/min (range, 0–120). One patient was inpatient and the others were outpatients.

**Table 1:** The clinical characteristics in the six patients

Case number	1	2	3	4	5	6
Age (years)	36	22	34	35	42	21
Gestational age in week	35	39	37	40	26	27
Gravidity	Primigravida	Primigravida	Primigravida	Primigravida	Primigravida	Primigravida
Height (cm)	147	157	148	161	160	147
Weight (kg)	77	78	61	54	44	48
Indication for C/S	Abruptio placenta	Abruptio placenta	Fetal distress	Abruptio placenta	Fetal distress	Fetal distress
Single or twin pregnancy	Single	Single	Single	Single	Single	Twin
FHR on arrival at our institution (beats/min)	110	22	64	88	90	120 (I) 0 (II)
Comorbidities						
Hypertension	+	−	−	−	+	−
Proteinuria	+	−	−	−	−	−
Hemodialysis	−	−	−	−	+	−
Clinical history of uterine surgery	+	−	−	−	−	−
In- or out-patient	Outpatient	Outpatient	Inpatient	Outpatient	Outpatient	Outpatient

*C/S*, cesarean section; *FHR*, fetal heart rate

The time course and surgical data were shown in Table 2. The four patients received the surgery at daytime, and the other two patients at nighttime. The average DDI was 21 min (range, 10–29). Maternal surgical complications did not occur, and they were discharged within the 9th postoperative day.

**Table 2:** The time course and surgical data of the six patients

	1	2	3	4	5	6-1	6-2
Time course							
Request for transfer to my institution	18:59	11:40	–	7:45	8:40	22:20	
Arrival at my institution	19:42	12:02	–	8:26	9:40	23:14	
Decision for cesarean section	19:42	12:02	8:25	8:26	9:40	23:14	
Arrival at the operation theater	19:57	12:05	8:45	8:39	9:52	23:22	
Start of the operation	20:08	12:12	8:53	8:42	9:58	23:31	
Delivery of the neonate	20:11	12:12	8:54	8:42	10:06	23:32	23:33
End of the operation	21:08	13:06	9:42	9:36	11:04	00:03	
DDI (min)	29	10	29	16	26	18	19
Surgical data							
Surgical duration (min)	60	54	49	54	66	32	
Intraoperative blood loss including amniotic fluid (ml)	876	1274	904	746	742	590	
Fluid infusion (ml)	1600	2000	1020	2210	1300	550	
Urine output (ml)	30	700	310	250	0	390	
Maternal surgical complications	–	–	–	–	–	–	
Discharge date of the mother	9th POD	5th POD	6th POD	6th POD	4th POD	7th POD	

*DDI* Decision-to-delivery interval, *POD* Postoperative day

The clinical characteristics and outcomes of the seven neonates were shown in Table 3. The mortality was 25% in the four neonates whose gestational ages of >35 weeks, and that was 67% in the three neonates whose gestational ages of <27 weeks. Except for one stillbirth, data of the residual six neonates were presented. Median 1st and 5th minute Apgar score was 1.5 (range, 1–3) and 2.5 (range, 2–6), respectively. The arterial cord blood analysis showed acidosis in the four neonates, and in the three of them, pH, base excess, and HCO_3_^−^ could not be measured accurately because of their too low values.

**Table 3:** The clinical characteristics and outcomes of the seven neonates

Case number	1	2	3	4	5	6-1	6-2
Outcome	Alive	Dead (Day 0 after birth)	Alive	Alive	Dead (Day 3 after birth)	Alive	Stillbirth
Fetal heart rate (beats/min)	110	22	64	88	90	120	0
Gestational age in week	35	39	37	40	26	27	
DDI (min)	29	10	29	16	26	18	19
Apgar score							
1st minute	1	2	3	1	1	2	0
5th minute	3	2	6	2	2	4	0
Birthweight (g)	1919	3068	2346	2306	598	805	Not measured
Findings of arterial cord blood test							
pH	<7.00	<7.00	7.36	<7.00	7.28	7.37	Not measured
Base excess (mmol/L)	Cannot be measured	Cannot be measured	− 0.8	Cannot be measured	− 17.8	− 0.6	Not measured
PaCO_2_ (mmHg)	69.0	78.0	44.0	72.0	13.0	43.0	Not measured
HCO_3_^−^ (mmol/L)	Cannot be measured	Cannot be measured	24.9	Cannot be measured	6.1	24.9	Not measured

*DDI* Decision-to-delivery interval

## Discussion

I considered that several factors might contribute to the achievement of the 30-min goal in all the subjects. First, one operating room was always kept empty for undergoing an obstetric emergency surgery. Second, preoperative written informed consent was skipped. Third, a simulation training for category-1 emergency C/S was held regularly, and we discussed the shortest route for transferring a patient to the operating theater. Fourth, human resources were available immediately. In our subjects, four patients received the surgery at daytime and two received at nighttime. It is noted that category-1 emergency C/S performed in the daytime had a statistically shorter DDI when compared with the nighttime (odds ratio=2.49, 95% confidence interval=1.26–4.92) [4]. Fortunately, the two patients received nighttime surgery were transferred to our institution soon after the other emergency surgery completed, and human resources were thus available easily. Fifth, general anesthesia was selected. Patients who received general anesthesia were 4 times more likely to achieve the 30-min goal than those who received regional anesthesia (odds ratio=4.0, 95% confidence interval=1.6–10.0) [4]. On the other hand, the use of general anesthesia for category-1 emergency C/S was 74.3% after the COVID-19 pandemic, which was significantly lower than that before the COVID-19 pandemic, at 86.6% (*p* value=0.037) [5]. The use of regional anesthesia increased significantly to decrease the risk of COVID-19 transmission to health care workers [5]. Furthermore, median DDI after and before the COVID-19 pandemic was 27 min and 26 min, respectively, and the difference was significant (*p* value=0.043) but was a little [5]. The appropriate anesthetic management in category-1 emergency cesarean section is controversial.

It is noted that DDI of <30 min does not always improve the neonatal outcomes [6]. The odds ratios (95% confidence intervals) on the incidence of adverse neonatal outcome were as follows: DDI, 0.957 (0.906–1.012); birthweight, 1.000 (1.0–1.001); male sex of newborn, 1.132 (0.744–1.722); diabetes mellitus or gestational diabetes, 0.791 (0.354–1.766); and gestational age, 0.869 (0.783–0.964) [7]. Indeed, in my study, the mortality in the neonates whose gestational ages of <27 weeks was higher compared to that in the neonates whose gestational ages of >35 weeks.

Furthermore, among the three patients with abruptio placenta, one neonate died despite the shortest DDI. In this case, abruptio placenta occurred during the induced labor, by using uterotonic agents and the fetal condition was nearly cardiac arrest. When abruptio placenta occurs, the neonatal mortality is reported to be approx. 30% [8], and the prognosis may depend on the severity of abruptio placenta rather than DDI.

The incidence of 5th minute Apgar score of <7 in the case of DDI was >60 min was 58.3%, which was significantly higher than that in the case of DDI was <30 min at 1.9% [9]. In my subjects, 5th minute Apgar score was <7; nevertheless, the 30-min goal was achieved. The cause is unknown; however, the average interval between request for the transfer of a patient and delivery of the neonate was 65 min (range, 32–87), which might be associated with the low 5th minute Apgar score. I thus consider that trying to shorten the time for transferring a patient is our future task to be solved.

## Conclusion

DDI in category-1 emergency C/S was achieved within 30 min in all the patients, and the mortality of neonate might depend on gestational age.

## Data Availability

The datasets analyzed during this study are available from the corresponding author on reasonable request.

## Abbreviations

C/S: Cesarean section
DDI: Decision-to-delivery interval
FHR: Fetal heart rate
